# Urinary extracellular vesicles and micro-RNA as markers of acute kidney injury after cardiac surgery

**DOI:** 10.1038/s41598-022-13849-z

**Published:** 2022-06-21

**Authors:** Douglas Miller, Bryony Eagle-Hemming, Sophia Sheikh, Lathishia Joel-David, Adewale Adebayo, Florence Y. Lai, Marius Roman, Tracy Kumar, Hardeep Aujla, Gavin J. Murphy, Marcin J. Woźniak

**Affiliations:** Department of Cardiovascular Sciences and NIHR Cardiovascular Biomedical Research Unit, Glenfield General Hospital, Clinical Sciences Wing, University of Leicester, Leicester, LE3 9QP UK

**Keywords:** Biomarkers, Biomarkers

## Abstract

We hypothesised that measuring changes in urinary levels of EV and miR will predict the onset of acute kidney injury in cardiac surgery patients. The study was performed in the cohort of the REVAKI-2 trial. Urine samples were collected before and 24 h after the procedure from 94 cardiac surgery patients. Urinary particle concentrations and size distribution were assessed using NanoSight. EV derivation and levels were measured using flow cytometry. Samples from 10 selected patients were sequenced, and verification was performed with advanced TaqMan assays in samples from all patients. Urinary particle concentrations significantly increased in patients with AKI after surgery, with the percentage of EV positive for CD105 and β1-integrin also increasing. Pre-surgery podocalyxin-positive EV were significantly lower in patients with AKI. Their levels correlated with the severity of the injury. Pre-operative miR-125a-5p was expressed at lower levels in urine from patients with AKI when adjusted for urinary creatinine. Levels of miR-10a-5p were lower after surgery in AKI patients and its levels correlated with the severity of the injury. Pre-operative levels of podocalyxin EVs, urinary particle concentrations and miR-125a-5p had moderate AKI predictive value and, in a logistic model together with ICU lactate levels, offered good (AUC = 82%) AKI prediction.

## Introduction

Acute kidney injury (AKI) is a common complication of cardiac surgery affecting up to 30% of patients. Patients who develop AKI suffer from increased morbidity and are four times more likely to die in hospital. Patients with AKI also need longer hospitalization, which increases healthcare costs^1–3^. AKI is defined by an acute drop in glomerular filtration rate as a result of injury to epithelial cells in renal tubules. Routine monitoring of the injury includes measuring urine output and the accumulation of circulating creatinine, a by-product of muscle metabolism. However, serum creatinine lacks diagnostic and prognostic accuracy, and the development of new and better biomarkers (e.g. Dickkopf^4^ and TIMP2/IGFBP7^5^) has been the subject of intensive research in recent years. However, the pathogenesis of AKI is still poorly understood, many novel and established biomarkers are affected by underlying conditions, and no biomarker has emerged as the AKI gold standard for diagnosis or risk stratification^6^.

Extracellular vesicles (EV) are lipid membrane-bound structures less than 1 µm in size. The EV are classified as microvesicles that bud off the plasma membrane or exosomes that are secreted through the exocytic pathway. EV contain proteins specific for their origin; therefore, changes in specific EV’s concentration during pathological processes can point towards affected cells or tissues (reviewed in Ref.^7^). In addition, EVs contain different types of RNA molecules that can influence gene expression in the target cells, and as such, are means of intracellular communication. These include messenger RNA, microRNA (miR) and other non-coding RNA. miRs are short (19–23) oligonucleotides that regulate post-transcriptional gene expression. Several of them contribute to AKI (reviewed in Ref.^8^), and some plasma derived miR have been shown to be AKI biomarkers in children^9^ and adults^10^.

We hypothesized that urinary EV and miR levels differ at baseline or post-cardiac surgery in people who progress to AKI. The aim of this study was to establish whether these markers may serve as novel predictive or diagnostic biomarkers of AKI.

## Results

**Figure 1:**
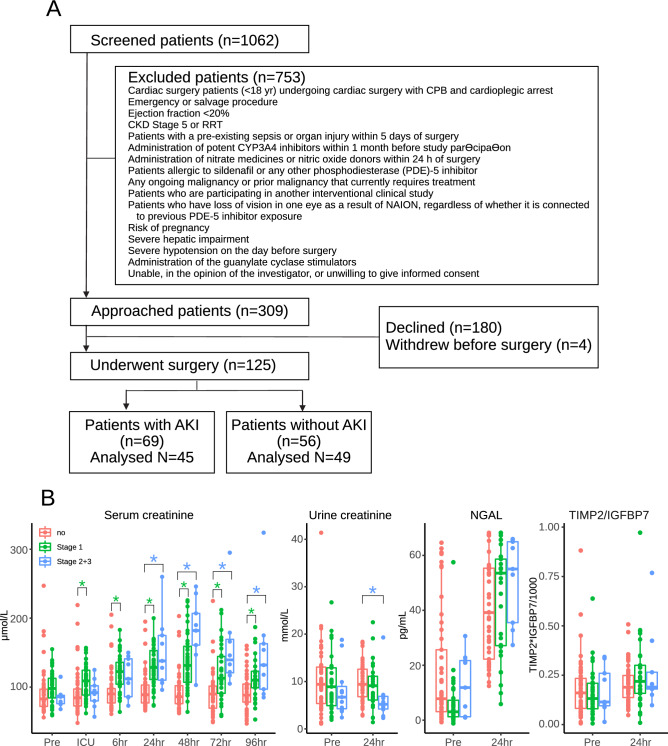
(**A**) CONSORT diagram. (**B**) Levels of serum creatinine and urinary creatinine, NGAL and TIMP2/IGFBP7. Green asterisks indicate a significant difference (*p* < 0.05) between no-AKI and AKI stage 1; blue asterisks indicate a significant difference between no-AKI and AKI stage 2/3.

Between September 2015 and September 2018, 1,062 patients undergoing cardiovascular surgery were screened for participation in the REVAKI-2 trial^11^. Three hundred nine were eligible, out of which 180 declined consent, resulting in 125 included patients. Sixty-nine patients developed post-surgery acute kidney injury, and 56 did not. The analysis population included 45 patients with AKI and 49 without (Fig. 1A). The median age was 74 (non-AKI) and 71 (AKI), of which 11 and 6% were females. The AKI groups were well matched apart from preoperative serum troponin I levels, which were higher in the AKI group. Patients with AKI also had significantly higher levels of lactate measured immediately after surgery, postoperative acute lung injury and multiple organ injury score. Levels of missing data were low, and less than 10% for all values but pre-operative troponin (Table 1). The most common was AKI stage 1 (n = 36), followed by stage 2 (n = 8) and stage 3 (n = 1).

**Table 1:** Pre- and Postoperative characteristics in the analyzed cohort.

n = 94		No AKI	AKI	p-value	Missing data (n)
Age (years)—median (IQR)		74 (70–78)	71 (66–75)	0.12	0
Sex (female)—n (%)		11 (11.7%)	6 (6.38%)	0.29	0
Ethnic (Caucasian)—n (%)		49 (52.13%)	43 (45.74%)	0.23	0
BMI		30.8 (27.6–33.4)	30.8 (25.5–37.3)	0.87	0
Sildenafil intervention		25 (26.6%)	20 (21.28%)	0.54	0
Diabetes—n (%)		14 (14.89%)	22 (23.4%)	0.06	0
Stroke/Transient Ischaemic Attack—n (%)		0 (0%)	1 (1.08%)	0.48	1
Chronic obstructive pulmonary disease—n (%)		4 (4.26%)	3 (3.19%)	1	0
Renal disease—n (%)		7 (7.29%)	0 (0%)	1	0
Myocardial infarction—n (%)		19 (19.79%)	0 (0%)	0.35	0
Pulmonary hypertension—n (%)		12 (12.5%)	0 (0%)	0.59	0
Anemia n (%)		21 (22.83%)	24 (26.09%)	0.4	2
Surgery type	CABG—n (%)	18 (19.15%)	17 (18.09%)	0.87	0
	Valve—n (%)	12 (12.77%)	14 (14.89%)		
	CABG & Valve—n (%)	15 (15.96%)	11 (11.7%)		
	other—n (%)	4 (4.26%)	3 (3.19%)		
NYHA	Class I—n (%)	6 (6.67%)	5 (5.56%)	0.95	4
	Class II—n (%)	33 (36.67%)	29 (32.22%)		
	Class III, IV—n (%)	8 (8.89%)	9 (10%)		
CCS	Asymptomatic—n (%)	17 (18.89%)	11 (12.22%)	0.7	4
	Class I—n (%)	13 (14.44%)	14 (15.56%)		
	Class II—n (%)	14 (15.56%)	16 (17.78%)		
	Class III, IV—n (%)	3 (3.33%)	2 (2.22%)		
Left Ventricular Ejection Fraction	Good(> 49%)—n (%)	38 (40.86%)	28 (30.11%)	0.13	1
	Fair(30–49%)—n (%)	9 (9.68%)	13 (13.98%)		
	Poor(< 30%)—n (%)	1 (1.08%)	4 (4.3%)		
Extent of coronary disease	Normal/ 1VD—n (%)	21 (22.58%)	23 (24.73%)	0.46	1
	2VD—n (%)	5 (5.38%)	7 (7.53%)		
	3VD—n (%)	22 (23.66%)	15 (16.13%)		
Pre-operative PaO2/FiO2 ratio—median (IQR)		457.14 (409.5–533.3)	457.14 (409.5–533.3)	0.7	2
Pre-operative Serum Creatinine (umol/L)—median (IQR)		83 (72–97)	92 (79–111)	0.07	0
Pre-operative eGFR—mean (STD)		76 (63.5–88)	69.6 (59–80)	0.27	0
eGFR < 60—n (%)		8 (8.51%)	14 (14.89%)	0.14	0
Pre-operative Serum Troponin (ng/mL)—median (IQR)		10 (7.8–20.4)	20 (10–36)	**0.01**	14
Pre-operative Serum NT-proBNP (pg/mL)—median (IQR)		6 (5.9–12.2)	10 (10.1–25.9)	0.31	4
Pre-operative MODS—median (IQR)		0 (0–1)	1 (0–1)	0.05	5
Pre-operative Lactate—median (IQR)		1 (0.8–1.5)	1.35 (1–1.63)	0.09	7
Pre-operative mean arterial pressure—median (IQR)		95.5 (91–105.5)	96.5 (88–104)	0.68	0
Post-operative Lactate (at return to ICU)—median (IQR)		1.9 (1.58–2.6)	2.6 (2.02–3.58)	** < 0.01**	3
Post-operative acute lung injury—mean (SD)		10 (10.64%)	19 (20.21%)	**0.03**	0
CBP time—median (IQR)		102 (72–122)	102 (86–134)	0.22	0
Cross-clamp time—median (IQR)		65 (46–80)	63 (52–85)	0.67	0

*ACE* angiotensin-converting enzyme, *AKI* acute kidney injury, *CABG* coronary artery bypass grafting, *CCS* Canadian Cardiovascular Society, *Hct* Hematocrit, *FiO2* fraction of inspired oxygen, *KDIGO* the kidney disease improving global outcomes, *MODS* multiple organ dysfunction score, *NYHA* New York Heart Association, *PO2* partial pressure of oxygen, *RBC* red blood cells, *VD* vessel disease.

(*) Tests between groups were conducted by exact test for categorical variables and ANOVA or non-parametric Kruskal–Wallis test for continuous variables. Data are presented as n (%) for categorical variables and mean (standard deviation, STD) or median (interquartile range, IQR) for continuous variables.

Significant values are given in bold.

Serum creatinine levels peaked 48 h after surgery and were highest in patients with AKI stages 2 and 3. Urinary creatinine displayed a reverse relationship 24 h after the surgery. There were no differences between AKI and non-AKI with respect to urinary levels of NGAL and TIMP2/IGFBP7, although concentrations of both markers increased 24 h after surgery (Fig. 1B).

**Figure 2:**
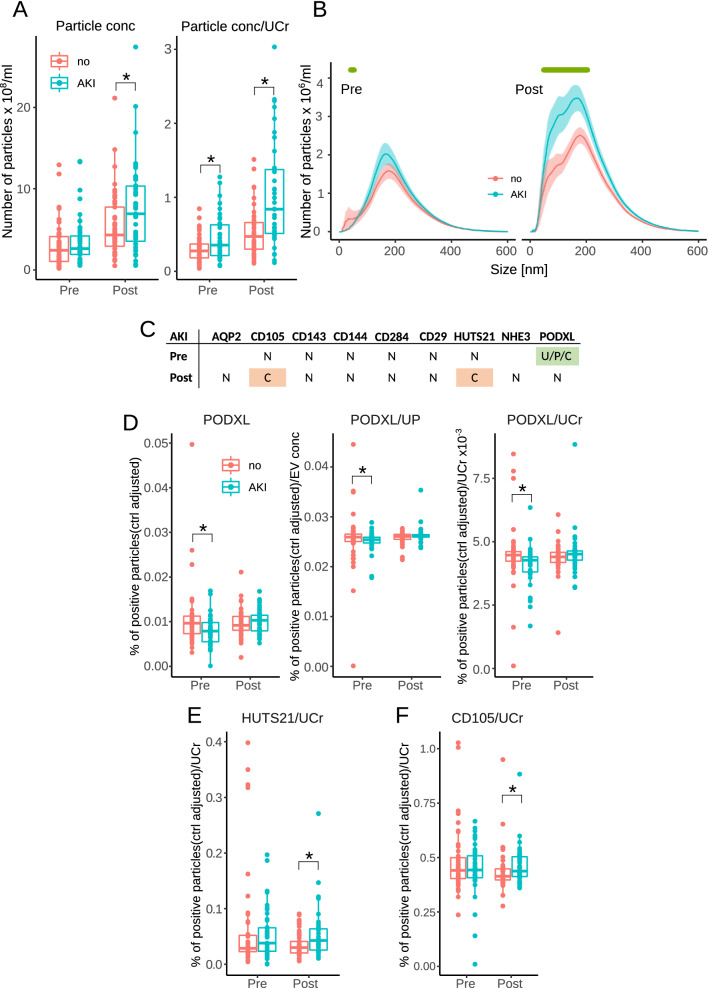
(**A**) Total particle concentrations in urine (unadjusted and urinary creatinine concentrations-adjusted). (**B**) Size distribution of urinary particle concentrations. Green bars indicate a significant difference between no-AKI and AKI at each particle size. (**C**) Summary of statistical analysis of EV using unadjusted (U), urinary particle (P) or urinary creatinine-adjusted (C) data. Green fields indicate a significant difference for all three adjustments and pink for one adjustment. N indicates no significant difference. (**D**) Plots of EV positive for PODXL, (**E**) activated β1-integrin (HUTS21 and (**F**) CD105. UCr indicates that the data were adjusted for urinary creatinine. Asterisks indicate a significant difference between AKI and non-AKI groups (*p* < 0.05).

Urinary particle concentrations were significantly different between AKI groups post-surgery. Urinary creatinine-adjusted particle concentrations were significantly different before and after surgery (Fig. 2A). When analysing AKI stages, we observed significant differences between no-AKI and stage 2 + 3 pre-surgery and between no-AKI and stage 1 and stage 2 + 3 post-surgery (Fig. S1A). Particles smaller than 200 nm were significantly more populous post-surgery in patients with AKI (Fig. 2B). That was also true when comparing no-AKI with AKI stage 1 (Fig. S1B). Particles greater than 300 nm and less than 100 nm in size were significantly more numerous in patients with AKI stages 2 and 3 post-surgery (Fig. S1B). Details of statistical analysis of urinary particles are in Table S1.

Flow analysis included exosomes positive for AQP2 (derived from collecting ducts^12^), NHE3 (expressed in renal proximal tubular cells^13^) and PODXL (specific for podocytes in the glomerulus^14^). Since flow cytometry is unreliable for exosomes analysis, the exosomes were first bound to CD81 beads, which were then tested with specific antibodies. Larger microvesicles were tested by direct binding to antibodies against β1-integrin (total—CD29, and activated—HUTS-21), endothelial and epithelial markers (CD105, CD143 and CD144) and toll-like receptor 4 (CD284). A summary of the unadjusted and adjusted EV analyses is shown in Fig. 2C. Levels of PODXL-positive exosomes were significantly lower in patients with AKI and were lowest in patients with stage 2/3. The differences were apparent in unadjusted and urinary particle or urinary creatinine-adjusted data (Figs. 2C,D and S1C,D). Patients with AKI had a significantly higher percentage of microvesicles positive for activated β1-integrin (adjusted for urinary creatinine concentrations, Fig. 2E) after surgery. The percentage of CD105-positive microvesicles, adjusted for urinary creatinine concentrations, was also significantly higher post-surgery in patients with AKI (Fig. 2F). Fractions of CD144-positive microvesicles were significantly higher before surgery in those who then developed post-operative stage 2/3 AKI in unadjusted and urinary creatinine concentrations-adjusted data (Fig. S2E). Finally, post-operative percentages of AQP2, activated β1-integrin and NHE3 vesicles adjusted for urinary creatinine were higher in patients with stage 2/3 AKI (Fig. S1F–H). Details of the statistical analysis of urinary EV are in Tables S2 and S3.

**Figure 3:**
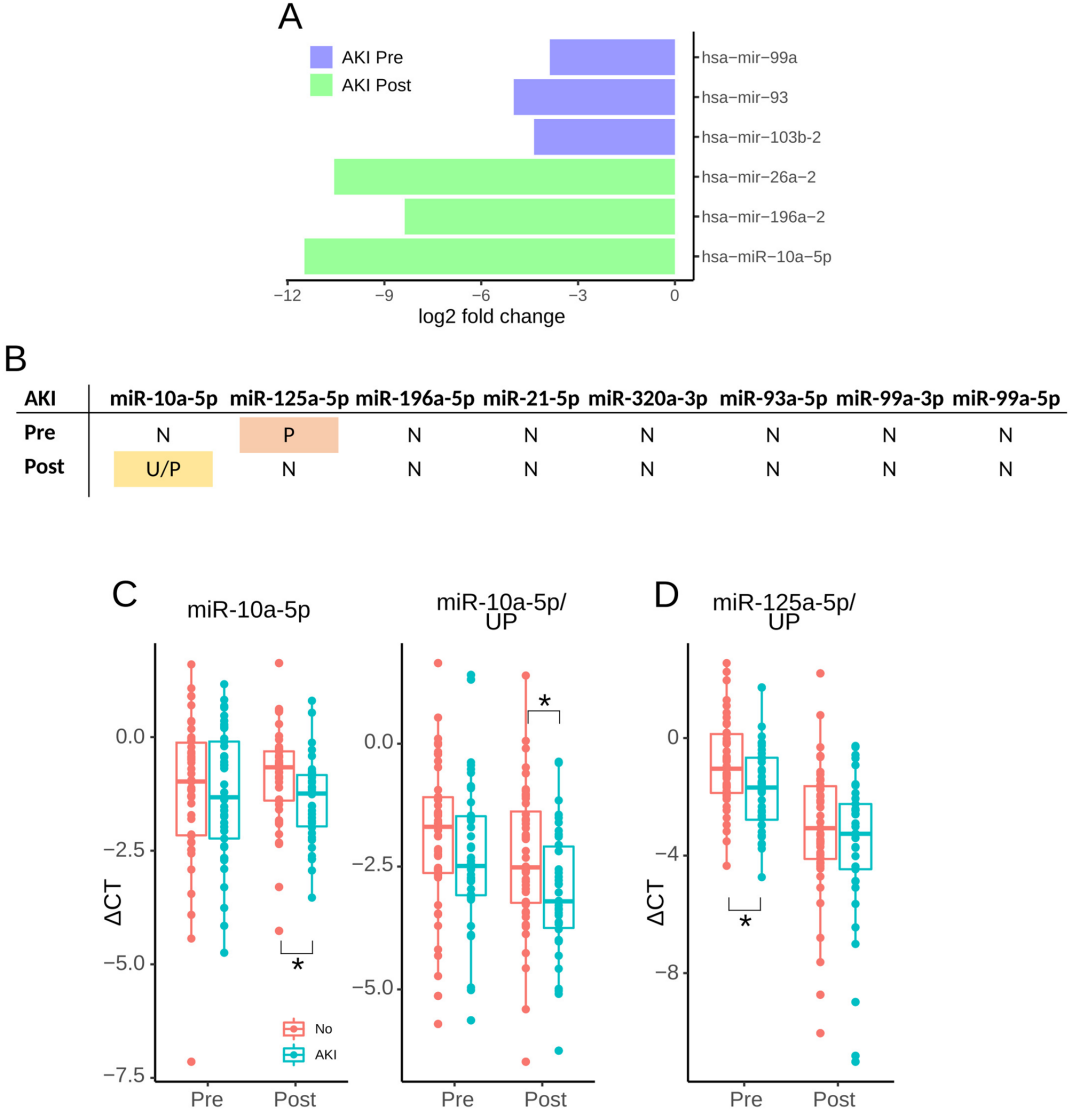
(**A**) Graphical representation of log2 fold changes of significantly different miRs. Negative values indicate lower expression levels in patients who developed AKI. (**B**) Summary of statistical analysis of qRT-PCR analysis of miR using unadjusted, EV or urinary creatinine-adjusted data. Yellow fields indicate a significant difference for two and pink for one adjustment. *U* unadjusted data, *P* urinary particle concentrations-adjusted data, *N* no difference. (**C**,**D**) Plots of miR-10a-5p and miR-125a-5p. Asterisks indicate a significant difference between AKI groups. UP indicates adjustment for urinary particle concentrations.

To identify AKI-specific miR, we sequenced RNA isolated from urinary extracellular vesicles. The analysis included five patients with AKI and five without. To avoid heterogeneity in such a small cohort, we only selected patients who developed AKI stage 1 and were without diabetes or CKD. Other characteristics were also well matched (Table S4). The sequencing data analysis identified six miR: miR-99a and 93 and 103b-2 were lower in AKI patients before surgery, whilst miR-26a-2, 196a-2 and 10a-5p we significantly lower in patients with AKI after surgery (Fig. 3A). Apart from mir-10a-5p, the sequencing analysis identified steam loops of miRs rather than mature miR. Therefore, in the following qRT-PCR verification, we included both mature strands for each miR steam loop. We also tested the expression of miR-125a-5p previously identified in children^9^ and miR-21, which is the most often reported urinary miR^15–17^. The list of all miRs tested in the whole cohort is in Table S5. Out of 13 planned miR measurements performed in all patients, only eight were detectable in the majority of the samples. miR-10a-5p and miR-125a-5p showed differential expression levels when comparing AKI groups (Fig. 3B). AKI patients had significantly lower levels of miR-10a-5p after surgery when unadjusted and urinary particle concentrations-adjusted (Fig. 3C) data were analysed. Post-surgery levels of miR-10a-5p decreased with AKI severity and were lowest in patients with stage 2/3 in urinary particle concentrations-adjusted data (Fig. S2B). Expression levels of miR-125a-5p were significantly lower before surgery in patients who developed AKI when analysing urinary particle concentrations adjusted data (Fig. 3D). The difference was likely due to AKI stage 1, as we did not detect a significant difference between no AKI and AKI stage 2 + 3 (Fig. S2C). Details of miR statistical analyses are in Tables S6 and S7.

**Figure 4:**
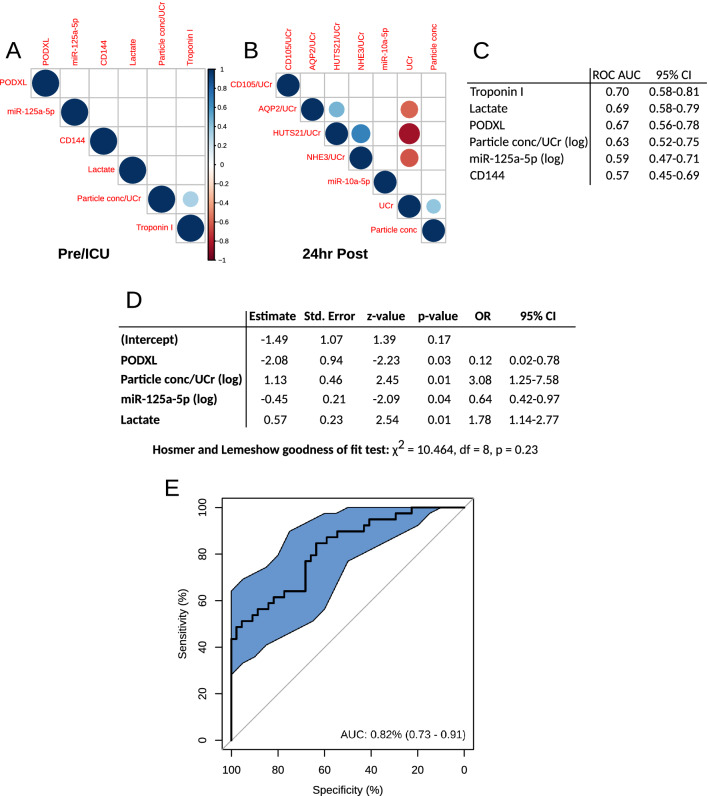
Correlations between biomarkers significantly different between AKI groups before or at the return from the theatre (**A**) and 24 h after surgery (**B**). (**C**) Predictive value of biomarkers that were significantly different before surgery. (**D**) Details of the logistic regression analysis. *OD* odds ratio. (**E**) ROC curve for a logistic model from (**D**).

Apart from urinary particle concentrations (adjusted for urinary creatinine levels) and troponin I, none of the five significantly different pre-operative variables and ICU lactate correlated with each other (Fig. 4A). Post-operatively, EV positive for AQP2 adjusted for urinary creatinine significantly correlated with urinary creatinine-adjusted EV positive for activated β1-integrin. Activated β1-integrin-positive EV adjusted for urinary creatinine significantly correlated with EV positive for NHE3, also adjusted for urinary creatinine. All three EVs correlated with urinary creatinine levels. Finally, urinary particle concentrations also correlated significantly with creatinine levels (Fig. 4B).

To assess the prognostic value of the pre-operative variables for AKI, we fitted a logistic regression model and calculated AUC for each of them. Troponin I, lactate, urinary particle concentrations adjusted for urinary creatinine and PODXL had AUC 0.63–0.70, whilst EV positive for CD144 and miR-125a-5p had AUC 0.57 and 0.59, respectively (Fig. 4C). We further fitted a multivariable logistic regression model with all these variables except troponin due to its high missing data levels and positive correlation with urinary particle concentrations. Levels of PODXL, miR-125a-5p, creatinine-adjusted urinary particles and lactate remained as significant predictors of AKI, whilst CD144 did not. In a model including only the significant variables, levels of PODX, creatinine-adjusted urinary particles and miR-125a-5p negatively correlated with the probability of AKI, whilst the lactate levels measured at the return to ICU correlated positively (Fig. 4D). Hosmer and Lemeshow test indicated that the model is of a good fit (p = 0.23) and has a very good predictive value for AKI (AUC = 0.82; Fig. 4E). We performed k-fold cross validation with 10 folds to further validate the model. The average accuracy between training and testing sets was 0.72 (SD = 0.12), and the average AUC for training sets was 0.82 (SD = 2.65; Fig. S3A). A model including only pre-operative variables (PODX, creatinine-adjusted urinary particles and miR-125a-5p) was also of good fit (Hosmer and Lemeshow p = 0.65) and had a good predictive value (AUC = 0.78; Fig. S3B). A model including ICU lactate only had AUC = 0.69% (Figs. 4C and S3C).

## Discussion

Levels of miR-125a-5p and EV derived from podocytes, epithelial cells, and expressing activated β1-integrin differed before surgery in patients with AKI. Out of these, only EV positive for PODXL urinary particle concentrations and miR-125a-5p were predictive for AKI. However, together with post-theatre lactate levels, their predictive value was excellent. After surgery, we observed increased concentrations of urinary particles accompanied by increases in EV positive for CD105 and activated β1-integrin adjusted for urinary creatinine concentrations. In addition, post-surgery levels of miR-10a-5p were lower in patients with AKI, and the decrease was proportional to the severity of AKI.

### Clinical importance

Kidney PODXL expresses specifically in podocytes, where it builds the glycocalyx. It is essential for podocyte differentiation and urine filtration^18^. We observed that levels of PODXL-positive exosomes were lower before surgery in patients who later developed AKI. The decreases were proportionate to the severity of AKI injury. Although the differences were small, they were apparent after adjusting for EV and urinary creatinine concentrations. That reassures us that the observed effect is of biological significance and can indicate a lower capacity of these patients’ kidneys to filter out waste products (e.g. creatinine), which lead to kidney injury after a surgical insult. PODXL is shed of podocytes together with apical membrane fragments in patients with nephritis^19^ and its increasing concentrations associate with glomerular damage^20^. These reports do not contradict our findings since exosomes are secreted from the exocytic compartment rather than bud-off from the plasma membrane. Instead, lower exosomal PODXL can indicate inefficient protein delivery to the plasma membrane or its lower expression levels in podocytes. Further experiments are necessary to verify this hypothesis.

We also observed increased levels of EV positive for activated β1-integrin and CD105 post-surgery but only when the data were adjusted for urinary creatinine concentrations. β1-integrin is expressed on most cells in its folded conformation. Its activation facilitates the adhesion of immune cells. Whether the observed rise in EV positive for β1-integrin correlates with inflammation levels needs further research. Levels of CD105 (endoglin) increase in response to AKI^21^ or ischaemia/reperfusion^22^ and can indicate endothelial activation.

Levels of NHE3, CD144 and activated β1-integrin (HUTS21) positive exosomes also increased after surgery but only in patients with AKI stage 2 or 3, indicating tubular damage^23^. AQP2 data (higher levels in AKI stage 2 or 3) is in contrast to some published reports where exosomal levels of aquaporins decrease during AKI. The decrease was associated with lower renal expression levels of aquaporins, leading to defects in water handling (reviewed in Ref.^24^). However, the reports come from animal models of AKI and may not reflect cardiac surgery-induced AKI. On the other hand, levels of AQP2-positive urinary exosomes increase in patients with diabetic nephropathy^12^. The AQP2 and activated β1-integrin results need to be taken with caution since the difference was only apparent after adjusting for urinary particle concentrations and can reflect changes in the total urinary particle concentrations.

Out of all tested miR, only two were statistically different in AKI patients. We previously reported miR-125a-5p to increase 6–12 h after surgery in plasma of children with AKI^9^. In this study, miR-125a-5p was expressed at higher levels pre-surgery in the urine patients who later developed AKI stage 2/3. It only has a predictive value for AKI when combined with podocalyxin and lactate, as its AUC was less than 0.60. However, it may be useful for more severe AKI, although we could not test it in our cohort since only nine patients developed stage 2 or 3. Levels of miR-10a-5p decreased after surgery, and the difference was evident in unadjusted and MV-adjusted data. There is not much evidence for the two identified miR as urinary markers of AKI. miR-10a-5p was previously reported as a circulatory marker of AKI, reaching exceptional predictive scores in a model with nine other miRs^25^. miR-21 previously reported in multiple papers as an AKI biomarker did not differ in our cohort.

We did not observe any difference in TIMP2/IGFBP7 and NGAL in patients with AKI as compared with non-AKI. The utility of TIMP2/IGFBP7 was established in patients admitted to intensive care units for various reasons, where cardiac surgery patients constituted only 8% of all subjects^5^. In addition, the number of AKI patients in the REVAKI-2 cohort was enriched using a modified AKI risk score that includes 15 factors significantly associated with post-cardiac surgery AKI. These include, among others, age, sex, BMI, diabetes, smoking status, hypertension, anaemia, ejection fraction, dyspnoea, glomerular filtration rate and the type of cardiac procedure. Compared with the Kashani et al. cohort, the REVAKI-2 cohort had a much lower percentage of females (6–11% vs 38%) and a lower percentage of patients with pre-operative glomerular filtration rate < 60 (8–14% vs 65%). Further investigation is needed to investigate whether these changes can explain the observed difference in the AKI biomarkers. Another explanation for the lack of difference could be that levels of TIMP2/IGFBP7 increase most in patients with AKI stage 2 or 3^5^ and there were only nine such patients in our cohort. Finally, AKI in the REVAKI-2 patients could be mainly due to a decreased capacity of podocytes to filter blood, which leads to alterations in glomerular haemodynamics rather than tubular damage. Levels of glomerular glycocalyx indeed decrease in response to cardiopulmonary bypass, which is associated with an increase in serum creatinine concentrations^26^. Low pre-operative PODXL-positive exosomes, absence of any difference in pre-operative NHE3-positive exosomes, and a significant increase in creatinine levels immediately after surgery in patients with stage 1 AKI support that hypothesis. Tubular damage likely happened in patients with AKI stages 2 and 3, forming 20% of AKI patients and not leading to noticeable increases in NGAL or TIMP2/IGFBP7.

### Limitations

The major limitation of studies into AKI biomarkers is using a suboptimal biomarker to define the injury. Serum creatinine can rise without damage to the kidney, and it is not sensitive enough to detect acute tubular injury^27^. Attempts have been made to redefine AKI using several recently discovered biomarkers like Dickkopf-3 (a predictive biomarker of AKI) or TIMP/IGFBP7 (markers of acute stress and damage) that also take into consideration serum creatinine and urinary output^28^. That would overcome detection difficulties of what appears like a heterogeneous injury that can be diagnosed only with a panel of biomarkers.

Another limitation is the small cohort of patients selected for sequencing analysis. The consequence was a small number of differentially expressed miRs, none of which passed false discovery rate adjustment. However, even despite that, miR-10a-5p appeared significantly different when measured in all patients using qRT-PCR. In addition, the sequencing cohort did not include patients who developed AKI stage 2–3, which biased our analysis towards AKI stage 1. The small number of patients probably increased the risk of overfitting the logistic model. Although the k-fold cross validation of the logistic model returned high accuracy, the model should be verified in a separate cohort of patients.

The study also suffers from flow cytometry limitations to detect EV subtypes^29^ even despite being the primary tool for the measurements. That limitation is true for microvesicle (CD144, CD284, HUTS-21 and CD29) analysis. Exosomes (PODXL, AQP2 and NHE3), on the other hand, were first bound to beads coupled to antibodies against CD81, which can be reliably recognised by flow cytometry.

The method used to estimate concentrations of urinary particles does not discriminate between membrane-bound vesicles and any other submicron particles. Our attempts to label the EV with a fluorescent marker failed as low levels of EV-bound fluorophores bleached too quickly. Moreover, no universal marker is present in all types of EV, e.g. exosomal markers (CD81, CD63 or CD9) are not present in microvesicles. Ultracentrifugation is another possibility to assess EV protein levels regardless of their origin; however, it is biased by the presence of protein aggregates. It also requires large amounts of urine, which were not available.

## Conclusions

Our results suggest that patients with existing damage to glomeruli are more likely to develop AKI after surgery. In the clinical settings of this study, the injury can be predicted using a model that includes urinary EV positive for PODXL, miR-125a-5p and urinary particle concentrations combined with lactate levels.

## Methods

### Study design

Predictive accuracy biomarker study performed in the cohort of the REVAKI-2 trial (registration ISRCTN18386427) that tested the effect of sildenafil (REVATIO®) on post-cardiac surgery acute kidney injury. The trial was a single centre parallel-group randomized clinical trial that has been published previously^11^. All participants in REVAKI-2 provided written informed consent to participate in the trial before their operation. The trial complied with the Declaration of Helsinki. The Yorkshire and The Humber Leeds East Research Ethics Committee approved the study (reference 15/YH/0489) on December 7, 2015. The University of Leicester was the trial sponsor.

### Study cohort

Study cohort included adult patients undergoing coronary artery bypass graft (CABG), open valve, or combined CABG and open-valve surgery who were at increased risk of developing AKI, as determined by a modified AKI risk score^1^ (a predicted risk score of 20% equates to a positive predicted value for developing AKI of > 55%) were eligible. Patients with pre-existing AKI, sepsis, Stage 5 chronic kidney disease (CKD), severe hepatic impairment, allergy to PDE Type 5 inhibitors, recent treatment with cytochrome P450 3A4 inhibitors or guanylate cyclase stimulators were excluded.

### Blood and urine sampling

Blood was collected before surgery and 6–12, 24, 48, 72 and 96 h after surgery (citrate and serum). Urine was collected before surgery and 24 h after.

### Outcomes

AKI and AKI stages were determined according to the Kidney Diseases Improving Global Outcomes (KDIGO) definition^30^. Other outcomes included plasma levels of troponin and NT-proBNP, urinary levels of TIMP2/IGFBP, NGAL, creatinine, total urinary particle concentrations, fractions of EV positive for podocalyxin, aquaporin 2, NHE3, CD143, CD144, activated β1-integrin (HUTS21) and CD29; levels of miR as defined by the sequencing analysis, and levels of selected miRs (Table S5) measured using real-time PCR technology. Multiple Organ Dysfunction Score (MODS) was calculated as previously described^31^.

### Biochemical biomarkers

Tissue metalloproteinase 2 (TIMP2) and insulin-like growth factor binding protein-7 (IGFBP7) were measured in urine using the human TIMP-2 Quantikine ELISA kit (Biotechne) and human IGFBP7 ELISA kit (abcam). Neutrophil gelatinase-associated lipocalin (NGAL) was measured in urine using Human Lipocalin-2/NGAL Quantikine ELISA Kit (BioTechne), Serum troponin was measured using High-Sensitivity Troponin I kit on Advia Centaur XP. Serum NT-proBNP was measured using NT-proBNP human ProcartaPlex™ Simplex kit (ThermoFisher) on the MAGPIX platform (Luminex).

### EV analysis

Urinary particle concentrations and size distributions were tested with NanoSight NS500 (Malvern Pananalytical) in 1 ml aliquots of urine diluted 1:50 with 0.2 µm filtered PBS.

#### Microvesicle analysis

Analysis was performed in 50 µl urine. All reagents (apart from antibodies) were filtered through 0.2 µm filters. Unspecific binding was blocked for 10 min with 15% BSA and 0.3 µg/ml human IgG. Antibody labelling was performed for 25 min at room temperature using 0.5–1 µg PE-coupled IgG against CD143 (clone 5-369, Biolegend), CD144 (clone 16Β1, Life Technologies/ThermoFisher), CD105 (clone MJ7/18, Life Technologies/ThermoFisher) and FITC-coupled CD284 (clone HTA2125, ThermoFisher), CD29 (TS2/16 clone, Life Technologies/ThermoFisher) and activated CD29 (HUTS-21 clone). For control, fluorophore-coupled isotype control IgGs were used (Life Technologies/ThermoFisher). Data acquisition was performed on CytoFLEX flow cytometer (Beckman Coulter) optimized with Submicron Bead Calibration kit (Polysciences).

#### Exosome analysis

Analysis was performed in 50 µl urine. Exosomes were first bound to CD81 magnetic beads (Invitrogen, ThermoFisher) and incubated for 30 min with 15% BSA and 0.3 µg/ml human IgG to block unspecific binding. Antibody labelling was performed for 25 min at room temperature with 0.5–1 µg IgG against aquaporin-2 (AQP2, clone E-2, Santa Cruz Biotechnology), podocalyxin (PODXL, polyclonal, Proteintech Europe) and sodium–hydrogen antiporter 3 (NHE3, clone 14D5, Insight Biotechnology) followed by incubation with Ax594-coupled secondary antibodies (Life Technologies/ThermoFisher). For controls, isotype control IgGs were used. Data acquisition was performed on CytoFLEX gated for CD81 beads.

#### miR analysis

EV’s RNA was isolated from 1 ml urine with an exoRNeasy kit (Qiagen) according to the manufacturer’s recommendations. Five microliters of the RNA preparation was used for sequencing library preparation using QIAseq miRNA Library kit (Qiagen). Library quality was assessed on BioAnalyzer 2100 with High Sensitivity DNA chip (Agilent). The presence of a peak of approximately 180 kb indicated successful library preparation. Libraries concentrations were calculated according to recommendations in the QIAseq kit. Sequencing (75 bp single reads) was performed on MiSeq (Illumina), aiming at a minimum of five million reads per sample.

The sequencing data were quality-checked with Fastqc v0.11.5^32^, aligned to miRBase 21^33^ using bowtie2^34^ as described in Ref.^35^ and quantified using Cufflinks^36^. FPKM quantities were analysed for differential expression using *limma* R-package.

Quantitative real-time PCR was carried on Qiagen’s Rotor-Gene machines using TaqMan Advanced miRNA assays (ThermoFisher) for miR-10a-5p, miR-125a-5p, miR-196a-5p, miR-320a-3p, miR-93a-5p, miR-99a-3p, miR-99a-5p and miR-21-5p. let-7a-5p was used as a reference control, since its levels did not change between the groups or over time.

### Statistical analysis

All data were analysed with R software for statistical computing and graphics^37^. Normally distributed continuous variables are reported as mean (standard deviation, SD). Variables not normally distributed are reported as median (interquartile range, IQR). Comparisons were performed between patients with and without AKI. The AKI patients were further split into Stage 1 and Stage 2 or 3 and compared with patients without AKI. Variable adjustments were performed by dividing the untransformed variable values by either urinary particle concentrations or urinary creatinine levels. Parametric comparisons between groups were performed using Welch’s t-test or ANOVA. Non-parametric comparisons were performed using Wilcox or Kruskal–Wallis test. All EV and miR data were analysed unadjusted and adjusted for either urinary creatinine or total urinary particle concentrations. Multivariable models for predicting AKI were built by binomial logistic regression. The goodness of fit was performed using the Hosmer and Lemeshow test. The area under the curve was calculated with *pROC* R-package. K-fold cross validation was performed as previously described^38^ for k = 10 each with 80/20 split between training and testing sets. The accuracy for each fold was calculated by predicting AKI in the testing data set using a model fit with the training data set and indicates a fraction of correct predictions.

## Supplementary Information

Supplementary Information.

## Data Availability

Sequencing and samples group data are available via NCBI Gene Expression Omnibus (GSE197272).

## References

[1] Birnie K (2014). Predictive models for kidney disease: Improving global outcomes (KDIGO) defined acute kidney injury in UK cardiac surgery. Crit. Care.

[2] Karkouti K (2009). Acute kidney injury after cardiac surgery: Focus on modifiable risk factors. Circulation.

[3] Lassnigg A (2004). Minimal changes of serum creatinine predict prognosis in patients after cardiothoracic surgery: A prospective cohort study. J. Am. Soc. Nephrol..

[4] Stefan JS (2019). Association between urinary dickkopf-3, acute kidney injury, and subsequent loss of kidney function in patients undergoing cardiac surgery: An observational cohort study. The Lancet.

[5] Kashani K (2013). Discovery and validation of cell cycle arrest biomarkers in human acute kidney injury. Crit. Care.

[6] Gayat E (2019). Biomarkers of acute kidney injury: Mixed results and huge heterogeneity of reporting. BMJ Evid. Based Med..

[7] Oh, S. & Kwon, S. H. Extracellular vesicles in acute kidney injury and clinical applications. *Int. J. Mol. Sci.* **22**, (2021). 10.3390/ijms22168913 PMC8396174 34445618

[8] Fan PC, Chen CC, Chen YC, Chang YS, Chu PH (2016). MicroRNAs in acute kidney injury. Hum Genomics.

[9] Sullo N (2018). An observational cohort feasibility study to identify microvesicle and micro-RNA biomarkers of acute kidney injury following pediatric cardiac surgery. Pediatr. Crit. Care Med..

[10] Fan PC (2019). A circulating miRNA signature for early diagnosis of acute kidney injury following acute myocardial infarction. J. Transl. Med..

[11] Kumar T (2020). Intravenous sildenafil citrate and post-cardiac surgery acute kidney injury: A double-blind, randomised, placebo-controlled trial. Br. J. Anaesth..

[12] Rossi L (2017). Urinary excretion of kidney aquaporins as possible diagnostic biomarker of diabetic nephropathy. J. Diabetes Res..

[13] Damien (2003). Urinary measurement of Na+/H+ exchanger isoform 3 (NHE3) protein as new marker of tubule injury in critically ill patients with ARF. Am. J. Kidney Dis..

[14] Pisitkun T, Shen RF, Knepper MA (2004). Identification and proteomic profiling of exosomes in human urine. Proc. Natl. Acad. Sci. U A.

[15] Saikumar J (2012). Expression, circulation, and excretion profile of microRNA-21, -155, and -18a following acute kidney injury. Toxicol. Sci..

[16] Du J (2013). MicroRNA-21 and risk of severe acute kidney injury and poor outcomes after adult cardiac surgery. PLoS ONE.

[17] Arvin P (2017). Early detection of cardiac surgeryassociated acute kidney injury by microRNA-21. Bratisl. Lek Listy.

[18] Nielsen JS, McNagny KM (2009). The role of podocalyxin in health and disease. J. Am. Soc. Nephrol..

[19] Hara M (2005). Apical cell membranes are shed into urine from injured podocytes: A novel phenomenon of podocyte injury. J. Am. Soc. Nephrol..

[20] Hara M (1995). Urinary excretion of podocalyxin indicates glomerular epithelial cell injuries in glomerulonephritis. Nephron.

[21] Szczepanski J (2020). Acute kidney injury during pregnancy leads to increased sFlt-1 and sEng and decreased renal T regulatory cells in pregnant rats with HELLP syndrome. Biol. Sex Differ..

[22] Docherty, N. G. *et al.* Endoglin regulates renal ischaemia-reperfusion injury. *Nephrol. Dial. Transplant. Off. Publ. Eur. Dial. Transpl. Assoc. - Eur. Ren. Assoc.* **21**, 2106–2119 (2006). 10.1093/ndt/gfl179 16751653

[23] du Cheyron, D. *et al.* Urinary measurement of Na+/H+ exchanger isoform 3 (NHE3) protein as new marker of tubule injury in critically ill patients with ARF. *Am. J. Kidney Dis. Off. J. Natl. Kidney Found.* **42**, 497–506 (2003). 10.1016/s0272-6386(03)00744-3 12955677

[24] Thongboonkerd V (2019). Roles for exosome in various kidney diseases and disorders. Front. Pharmacol..

[25] Aguado-Fraile E (2015). A pilot study identifying a set of MicroRNAs as precise diagnostic biomarkers of acute kidney injury. PLoS ONE.

[26] Qureshi SH, Pate NN, Murphy GJ (2018). Vascular endothelial cell changes in postcardiac surgery acute kidney injury. Am. J. Physiol. Renal Physiol..

[27] Waikar SS, Betensky RA, Emerson SC, Bonventre JV (2012). Imperfect gold standards for kidney injury biomarker evaluation. J. Am. Soc. Nephrol..

[28] Ostermann M (2020). Recommendations on acute kidney injury biomarkers from the acute disease quality initiative consensus conference: A consensus statement. JAMA Netw. Open.

[29] van der Pol E (2010). Optical and non-optical methods for detection and characterization of microparticles and exosomes. J. Thromb. Haemost.

[30] Kidney Disease: Improving Global Outcomes (KDIGO) Acute Kidney Injury Work Group. KDIGO Clinical Practice Guideline for Acute Kidney Injury. *Kidney Inter Suppl* **2**, 1–138 (2012).

[31] Marshall JC (1995). Multiple organ dysfunction score: A reliable descriptor of a complex clinical outcome. Crit. Care Med..

[32] Simon Andrews. FastQC: a quality control tool for high throughput sequence data.

[33] Kozomara A, Birgaoanu M, Griffiths-Jones S (2019). miRBase: From microRNA sequences to function. Nucleic Acids Res..

[34] Langmead B, Salzberg SL (2012). Fast gapped-read alignment with Bowtie 2. Nat. Methods.

[35] Tam S, Tsao MS, McPherson JD (2015). Optimization of miRNA-seq data preprocessing. Brief. Bioinform..

[36] Trapnell C (2010). Transcript assembly and quantification by RNA-Seq reveals unannotated transcripts and isoform switching during cell differentiation. Nat. Biotechnol..

[37] {R Core Team}. *R: A Language and Environment for Statistical Computing*. (R Foundation for Statistical Computing, 2018).

[38] Lever J, Krzywinski M, Altman N (2016). Model selection and overfitting. Nat. Methods.

