# Supplementary material for: Urinary extracellular vesicles and micro-RNA as markers of acute kidney injury after cardiac surgery
Source: Sci Rep. 2022 Jun 21;12:10402. doi: 10.1038/s41598-022-13849-z (PMC9213448; doi:10.1038/s41598-022-13849-z)
Supplement: Supplementary file 1 — Supplementary Information. [file 41598_2022_13849_MOESM1_ESM.pdf]

SUPPLEMENTAL MATERIAL

Urinary extracellular vesicles and micro-RNA as markers of acute kidney injury after cardiac surgery

Douglas Miller, Bryony Eagle-Hemming, Sophia Sheikh, Lathishia Joel-David, Adewale Adebayo, Florence Y Lai, Marius Roman, Tracy Kumar, Hardeep Aujla, Gavin J Murphy, Marcin J Woźniak

TABLE OF CONTENTS

Figure S1....................2
Figure S2....................3
Figure S3....................4
Table S1 – Summary of urinary particle concentrations comparisons....................5
Table S2 – Summary of urinary particle comparisons between AKI and non-AKI groups....................6
Table S3 – Summary of urinary particle comparisons between AKI stages....................8
Table S4 – Pre- and Postoperative characteristics in the sequenced cohort....................10
Table S5 – miR selection for verification with qRT-PCR....................11
Table S6 – Summary of miR comparisons between AKI and non-AKI....................12
Table S7 – Summary of miR comparisons between AKI stages....................14

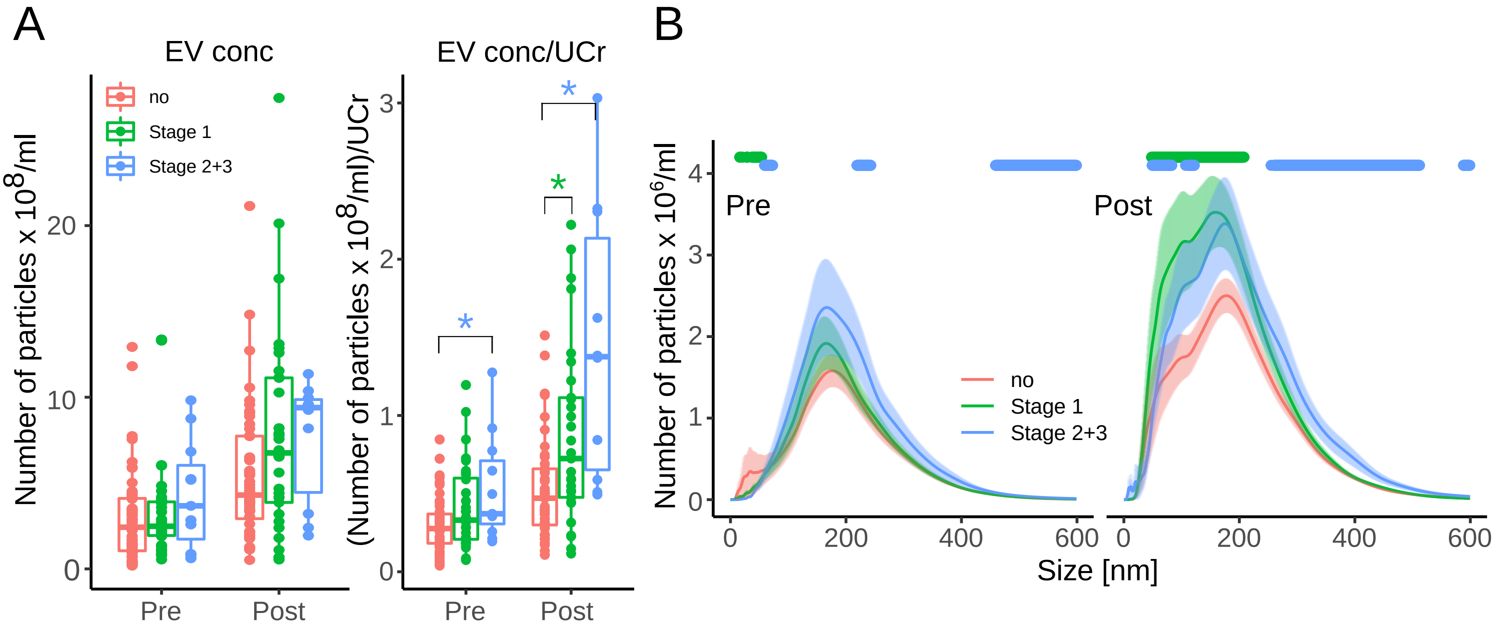

C

AKI stages	AQP2	CD105	CD143	CD144	CD284	CD29	HUTS21	NHE3	PODXL
Pre	N	N	N	U/C	N	N	N	N	U/C
Post	C	N	N	N	N	N	C	C	N

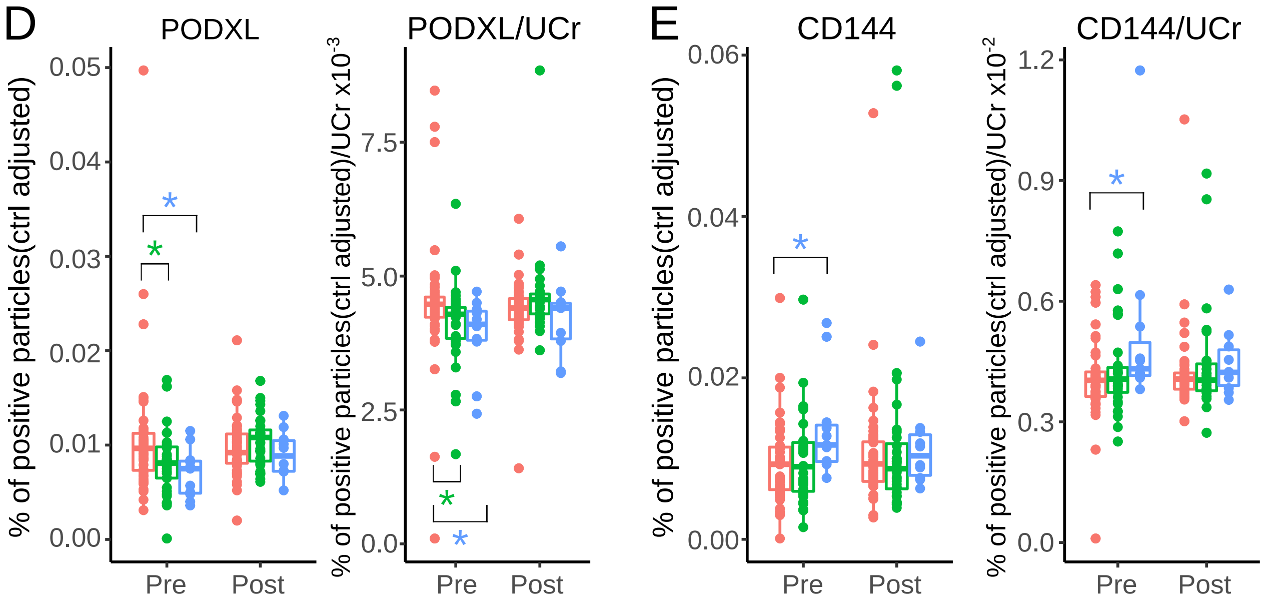

Figure S1 – (**A**) Total particle concentrations in urine (unadjusted and urinary creatinine concentrations-adjusted). (**B**) Size distribution of urinary particle concentrations. Green bars indicate a significant difference between no-AKI and AKI stage 1. Blue bars indicate a significant difference betwen no-AKI and AKI stage 2 or 3. (**C**) Summary of statistical analysis of EV using unadjusted (U) or urinary creatinine-adjusted (C) data. Yellow fields indicate a significant difference for two adjustments and pink for one adjustment. N indicates no significant difference. (**D**) Plots of EV positive for PODXL, (**E**) AQP2, (**F**) activated β1-integrin (HUTS21) and (**G**) NHE3. UCr indicates that the data were adjusted for urinary creatinine.

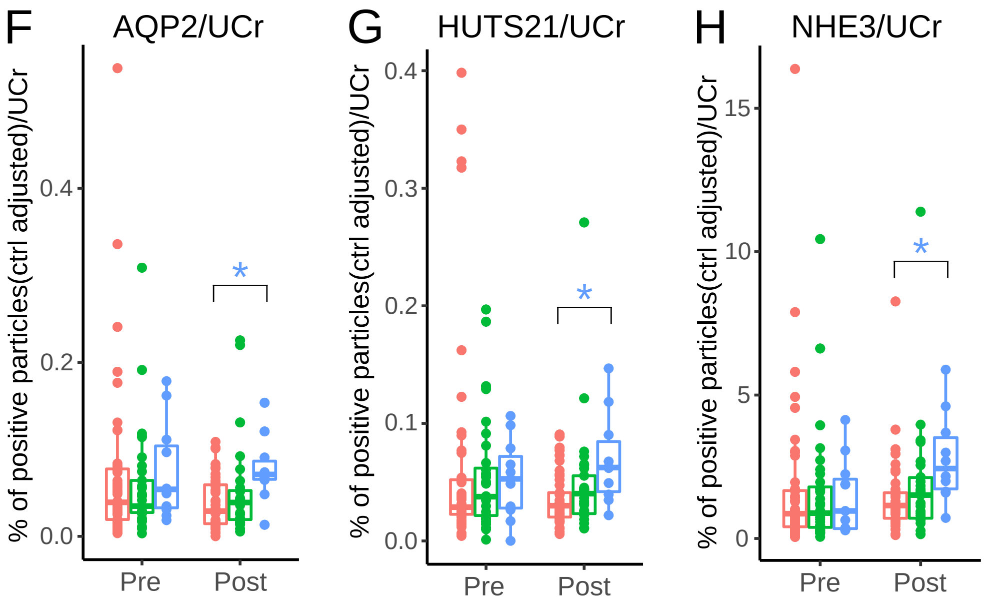

A

AKI stages	miR-10a-5p	miR-125a-5p	miR-196a-5p	miR-21-5p	miR-320a-3p	miR-93a-5p	miR-99a-3p	miR-99a-5p
**Pre**	N	U/P	N	N	N	N	N	N
**Post**	P	N	N	N	N	N	N	N

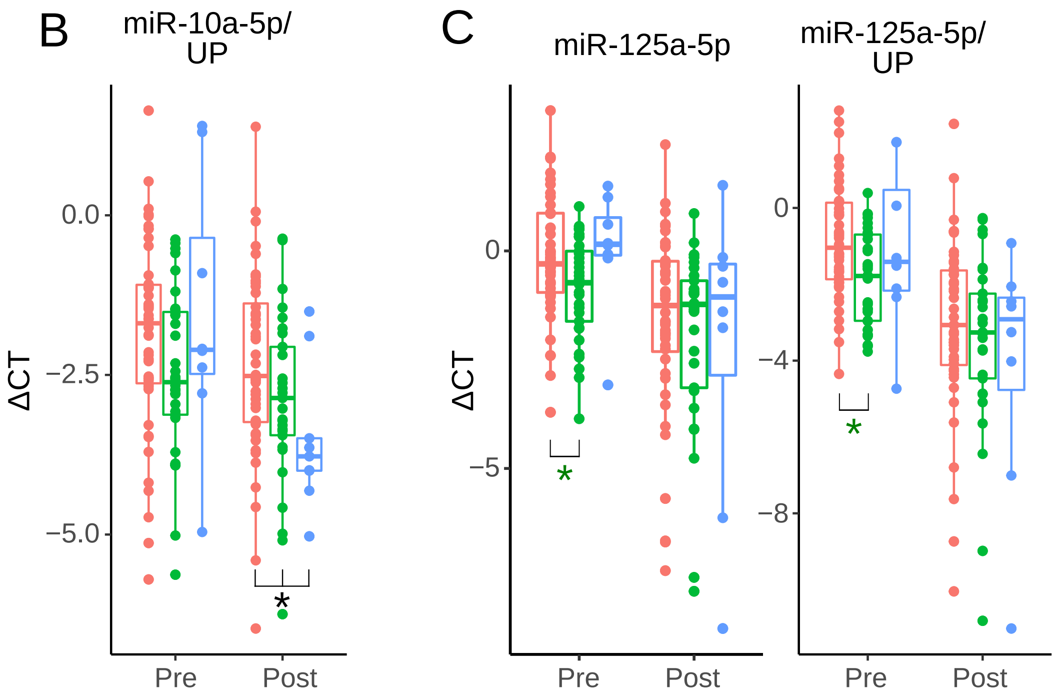

Figure S2 – (**A**) Summary of statistical analysis of qRT-PCR analysis of miR using unadjusted or urinary particle concentrations-adjusted data. Yellow fields indicate a significant difference for two and pink for one adjustment. U – unadjusted data, P – urinary particle concentrations-adjusted data, N – no difference. (**B – C**) Plots of miR-10a-5p and miR-125a-5p. Asterisks indicate a significant difference between AKI groups. UP indicates adjustment for urinary particle concentrations.

A

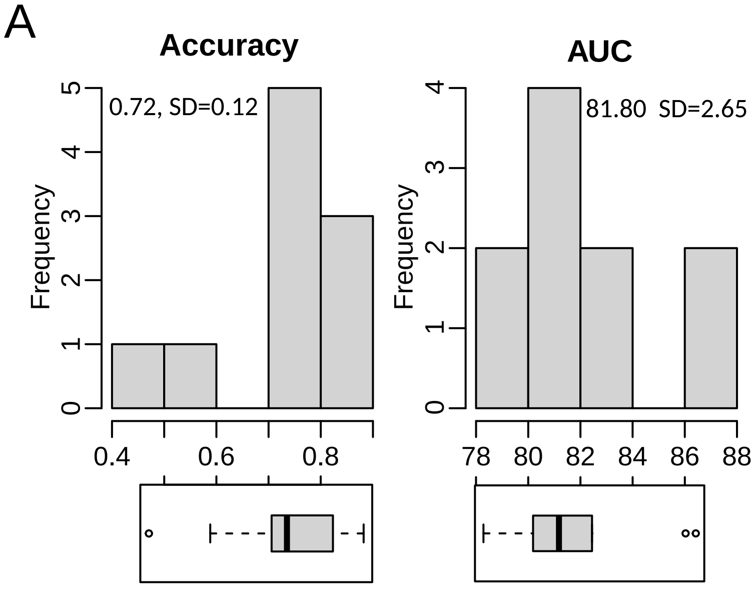

B

Pre: Podocalyxin + miR-125a-5p + EVconc/UCr

	Estimate	Std. Error	z-value	p-value	OR	95% CI
**(Intercept)**	2.88	0.90	3.19	<0.01		
**PODXL**	-2.02	0.83	-2.42	0.02	0.13	0.03-0.68
**miR-125a-5p (log)**	-0.41	0.20	-2.06	0.04	0.66	0.45-0.98
**EVconc/UCr (log)**	1.14	0.43	2.63	0.01	3.12	1.34-7.27

Hosmer and Lemeshow goodness of fit test: χ^2 = 5.98, df = 8, p = 0.65

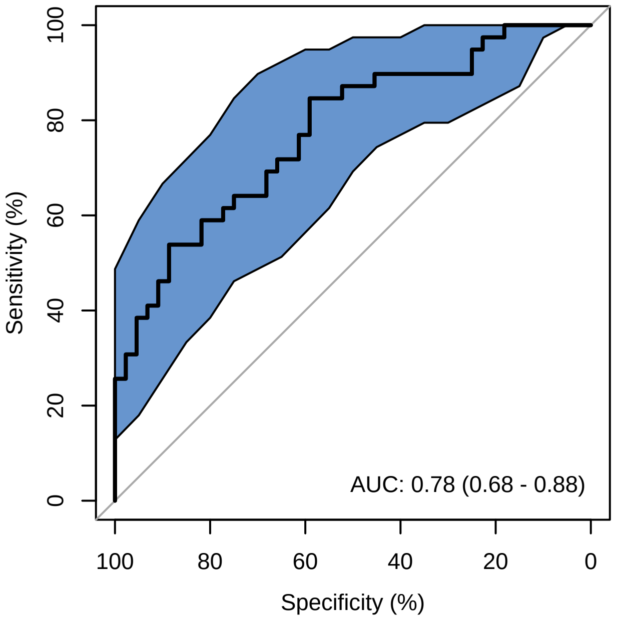

C

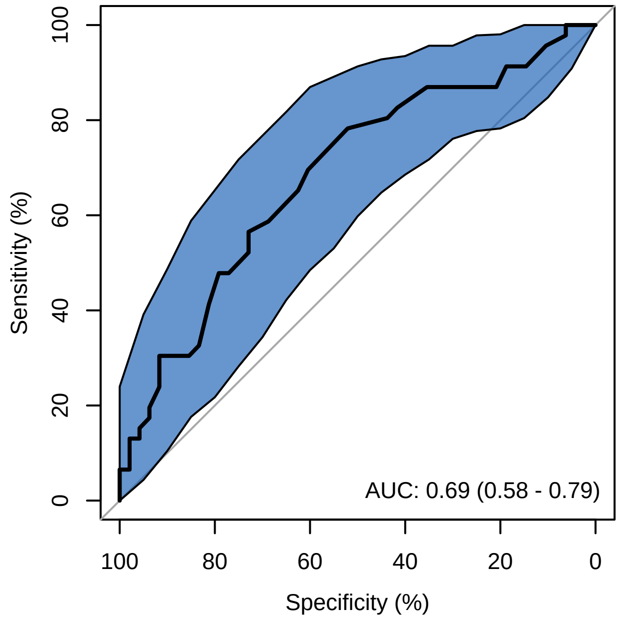

ICU Lactate

	Estimate	Std. Error	z-value	p-value	OR	95% CI
**(Intercept)**	-1.46	0.55	-2.73	0.01		
**Lactate**	0.53	0.20	2.60	0.01	1.70	1.14-2.54

Hosmer and Lemeshow goodness of fit test: χ^2 = 7.85, df = 8, p = 0.45

Figure S3 – (**A**) Summary of the k-fold cross validation (k=10). Histograms of accuracy frequencies between training and testing sets, and AUC frequencies for the training sets. Below histograms are boxplots of accuracies and AUC in the ten folds of the analysis. (**B**) Details of the logistic regression model including levels of PODXL-positive exosomes, miR-125a-5p and creatinine-adjusted urinary particle concentrations and its ROC curve. (**C**) Details of the logistic regression model including levels of ICU lactate and its ROC curve. OD – odds ratio.

Table S1 – Summary of urinary particle concentrations comparisons.

AKI vs non-AKI: top four rows, AKI stages: bottom four rows. Post hoc comparisons (Tukey or Dunn tests for parametric and non-parametric data, respectively): S1-no – AKI stage 1 vs no AKI, S2+S3-no – AKI stage 2 or 3 vs no AKI, S2+S3-S1 – AKI stage 2 or 3 vs AKI stage 1.

Time	EV	Transformation	Normal distr.	Test	p.value	S1-no	S2+S3-no	S2+S3-S1
Pre	EVconc	log	yes	t-test	0.11			
Post	EVconc	None	no	Wilcox	**0.02**			
Pre	Evconc*Ucr	log	yes	t-test	**0.01**			
Post	Evconc*Ucr	log	yes	t-test	**<0.01**			
Pre	EVconc	log	yes	ANOVA	0.24	0.41	0.34	0.86
Post	EVconc	none	no	Kruskal	0.06	0.09	0.05	0.29
Pre	Evconc*Ucr	log	yes	ANOVA	**0.02**	0.15	**0.03**	0.42
Post	Evconc*Ucr	log	yes	ANOVA	**0.00**	**0.02**	**<0.01**	0.06

Table S2 – Summary of urinary particle comparisons between AKI and non-AKI groups.

q-value – p-values adjusted for multiple comparison using Benjamini Hochberg method.

Adjustment	Time	EV	Transformation	Normal distr.	Test	p-value	q-value
None	Pre	AQP2	None	no	Wilcox	0.87	0.98
		CD105	None	no	Wilcox	0.60	0.91
		CD143	None	no	Wilcox	0.27	0.61
		CD144	None	no	Wilcox	0.26	0.61
		CD284	None	no	Wilcox	1.00	1.00
		CD29	None	no	Wilcox	0.43	0.77
		HUTS21	None	no	Wilcox	0.10	0.46
		NHE3	None	no	Wilcox	0.82	0.98
		PODXL	None	no	Wilcox	**0.00**	**0.04**
	Post	AQP2	None	yes	ttest	0.61	1.00
		CD105	None	no	Wilcox	0.36	0.95
		CD143	None	no	Wilcox	0.36	0.95
		CD144	None	no	Wilcox	0.84	1.00
		CD284	log	yes	ttest	1.00	1.00
		CD29	None	no	Wilcox	0.99	1.00
		HUTS21	None	no	Wilcox	0.42	0.95
		NHE3	None	no	Wilcox	0.90	1.00
		PODXL	None	no	Wilcox	0.35	0.95
Urinary particles concentrations	Pre	AQP2	log	yes	ttest	0.43	0.55
		CD105	None	no	Wilcox	0.24	0.53
		CD143	None	no	Wilcox	0.35	0.53
		CD144	None	no	Wilcox	0.33	0.53
		CD284	None	no	Wilcox	0.22	0.53
		CD29	None	no	Wilcox	0.81	0.81
		HUTS21	None	no	Wilcox	0.54	0.60
		NHE3	log	yes	ttest	0.28	0.53
		PODXL	None	no	Wilcox	**0.04**	0.34
	Post	AQP2	None	no	Wilcox	0.31	0.47
		CD105	None	no	Wilcox	0.83	0.93
		CD143	None	no	Wilcox	0.21	0.47
		CD144	None	no	Wilcox	0.89	0.93
		CD284	log	yes	ttest	0.12	0.47
		CD29	None	no	Wilcox	0.93	0.93

		HUTS21	log	yes	ttest	0.29	0.47
		NHE3	log	yes	ttest	0.21	0.47
		PODXL	None	no	Wilcox	0.12	0.47
Urinary creatinine levels	Pre	AQP2	log	yes	ttest	0.63	0.96
		CD105	None	no	Wilcox	0.91	0.96
		CD143	None	no	Wilcox	0.32	0.73
		CD144	None	no	Wilcox	0.16	0.71
		CD284	None	no	Wilcox	0.96	0.96
		CD29	None	no	Wilcox	0.77	0.96
		HUTS21	None	no	Wilcox	0.24	0.73
		NHE3	log	yes	ttest	0.82	0.96
		PODXL	None	no	Wilcox	**<0.01**	**0.04**
	Post	AQP2	None	no	Wilcox	0.09	0.19
		CD105	None	no	Wilcox	**<0.05**	0.19
		CD143	None	no	Wilcox	0.26	0.29
		CD144	None	no	Wilcox	0.51	0.51
		CD284	log	yes	ttest	0.24	0.29
		CD29	None	no	Wilcox	0.18	0.29
		HUTS21	log	yes	ttest	**0.02**	0.17
		NHE3	log	yes	ttest	0.07	0.19
		PODXL	None	no	Wilcox	0.25	0.29

Table S3 – Summary of urinary particle comparisons between AKI stages.

Post hoc comparisons (Tukey or Dunn tests for parametric and non-parametric data, respectively): S1-no – AKI stage 1 vs no AKI, S2+S3-no – AKI stage 2 or 3 vs no AKI, S2+S3-S1 – AKI stage 2 or 3 vs AKI stage 1.

Adjustment	Time	EV	Transformation	Normal distr.	Test	p-value	S1-no	S2+S3-no	S2+S3-S1
None	Pre	AQP2	none	no	Kruskal	0.81	0.52	0.35	0.79
		CD105	none	no	Kruskal	0.72	0.71	0.44	0.41
		CD143	none	no	Kruskal	0.27	0.29	0.16	0.17
		CD144	none	no	Kruskal	**0.04**	0.43	**0.02**	0.02
		CD284	none	no	Kruskal	0.61	0.35	0.33	0.48
		CD29	none	no	Kruskal	0.43	0.36	0.29	0.23
		HUTS21	none	no	Kruskal	0.15	0.20	0.09	0.14
		NHE3	none	no	Kruskal	0.93	0.46	1.00	0.58
		PODXL	none	no	Kruskal	**0.01**	**0.02**	**0.02**	0.16
	Post	AQP2	None	yes	ANOVA	0.54	0.99	0.51	0.58
		CD105	none	no	Kruskal	0.64	0.66	0.35	0.42
		CD143	none	no	Kruskal	0.27	0.36	0.16	0.14
		CD144	none	no	Kruskal	0.55	0.28	0.35	0.42
		CD284	log	yes	ANOVA	1.00	1.00	1.00	1.00
		CD29	none	no	Kruskal	1.00	0.73	0.49	1.00
		HUTS21	none	no	Kruskal	0.62	0.29	0.52	0.43
		NHE3	none	no	Kruskal	0.79	0.45	0.40	0.75
		PODXL	none	no	Kruskal	0.24	0.12	0.29	0.24
Urinary particle concentration	Pre	AQP2	log	yes	ANOVA	0.73	0.78	0.82	0.99
		CD105	none	no	Kruskal	0.47	0.33	0.50	0.35
		CD143	none	no	Kruskal	0.57	0.38	0.50	0.31
		CD144	none	no	Kruskal	0.17	0.39	0.09	0.08
		CD284	none	no	Kruskal	0.36	0.24	0.43	0.34
		CD29	none	no	Kruskal	0.58	0.44	0.26	0.46
		HUTS21	none	no	Kruskal	0.48	0.43	0.35	0.23
		NHE3	log	yes	ANOVA	0.49	0.71	0.52	0.85
		PODXL	none	no	Kruskal	0.11	0.06	0.26	0.33
	Post	AQP2	none	no	Kruskal	0.60	0.52	0.40	0.50
		CD105	none	no	Kruskal	0.97	1.00	0.50	0.66
		CD143	none	no	Kruskal	0.31	0.20	0.22	0.29
		CD144	none	no	Kruskal	0.88	0.56	0.38	0.94
		CD284	log	yes	ANOVA	0.29	0.36	0.49	0.97
		CD29	none	no	Kruskal	0.99	1.00	0.49	0.72

		HUTS21	log	yes	ANOVA	0.56	0.62	0.73	0.99
		NHE3	log	yes	ANOVA	0.45	0.44	0.80	0.99
		PODXL	none	no	Kruskal	0.22	0.12	0.35	0.35
Urinary creatinine concentrations	Pre	AQP2	log	yes	ANOVA	0.58	1.00	0.56	0.62
		CD105	none	no	Kruskal	0.79	0.44	0.41	0.75
		CD143	none	no	Kruskal	0.44	0.28	0.31	0.31
		CD144	none	no	Kruskal	**0.04**	0.32	**0.02**	**0.03**
		CD284	none	no	Kruskal	0.34	0.30	0.18	0.21
		CD29	none	no	Kruskal	0.46	0.42	0.19	0.33
		HUTS21	none	no	Kruskal	0.46	0.27	0.42	0.33
		NHE3	log	yes	ANOVA	0.94	0.99	0.94	0.97
		PODXL	none	no	Kruskal	**0.01**	**0.01**	**0.03**	0.23
	Post	AQP2	none	no	Kruskal	**0.01**	0.27	**<0.01**	**0.01**
		CD105	none	no	Kruskal	0.13	0.12	0.11	0.40
		CD143	none	no	Kruskal	0.35	0.25	0.24	0.28
		CD144	none	no	Kruskal	0.50	0.41	0.36	0.25
		CD284	log	yes	ANOVA	0.22	0.83	0.19	0.40
		CD29	none	no	Kruskal	0.40	0.30	0.34	0.45
		HUTS21	log	yes	ANOVA	**0.01**	0.30	**0.01**	0.13
		NHE3	log	yes	ANOVA	**0.02**	0.66	**0.02**	0.09
		PODXL	none	no	Kruskal	0.11	0.12	0.26	0.06

Table S4 – Pre- and Postoperative characteristics in the sequenced cohort.
(*) - Tests between groups were conducted by exact test for categorical variables and ANOVA or non-parametric Kruskal-Wallis test for continuous variables. Data are presented as n (%) for categorical variables and mean (standard deviation, STD) or median (interquartile range, IQR) for continuous variables.
Abbreviations: ACE – Angiotensin-Converting Enzyme; AKI – Acute Kidney Injury; CABG – Coronary artery Bypass Grafting; CCS – Canadian Cardiovascular Society; Hct – Hematocrit; FiO2 – Fraction of Inspired Oxygen; KDIGO - The Kidney Disease Improving Global Outcomes; MODS – Multiple Organ Dysfunction Score; NYHA – New York Heart Association; PO2 – Partial Pressure of Oxygen; RBC – Red Blood Cells; VD – Vessel Disease.

**n = 10**		**No AKI n=5**	**AKI n=5**	**p-value**	**Missing data (n)**
Age (years) - Mean (SD)		71 (7.55)	71.2 (3.35)	0.96	0
Sex (female) - n (%)		2 (20%)	1 (10%)	1	0
Ethnic (Caucasian) - n (%)		5	5	1	0
BMI - Mean (SD)		31.46 (3.61)	30.7 (7)	0.84	0
Sildenafil intervention		2 (20%)	1 (%)		
Diabetes - n (%)		0	0	NA	0
Stroke/Transient Ischaemic Attack - n (%)		0	0	NA	0
Chronic obstructive pulmonary disease - n (%)		0	0	NA	
Renal disease - n (%)		0	0	NA	0
Myocardial infarction - n (%)		0	0	NA	0
Pulmonary hypertension - n (%)		0	0	NA	0
Anemia n (%)		3 (30%)	1 (%)	0.52	
Surgery type	CABG - n (%)	3 (30%)	1 (%)	0.71	0
	Valve - n (%)	0 (0%)	1 (%)		
	CABG & Valve - n (%)	2 (20%)	2 (%)		
	other - n (%)	0 (0%)	1 (%)		
NYHA	Class I - n (%)	0 (0%)	1 (%)	1	0
	Class II - n (%)	3 (33.33%)	3 (%)		
	Class III, IV - n (%)	1 (11.11%)	1 (%)		
CCS	Asymptomatic - n (%)	0 (0%)	2 (%)	0.68	0
	Class I - n (%)	3 (33.33%)	2 (%)		
	Class II - n (%)	1 (11.11%)	1 (%)		
Left Ventricular Ejection Fraction	Good(>49%) - n (%)	5 (50%)	4 (%)	1	0
	Fair(30-49%) - n (%)	0 (0%)	1 (%)		
Extent of coronary disease	Normal/ 1VD - n (%)	2 (20%)	3 (%)	1	0
	2VD - n (%)	1 (10%)	0 (%)		
	3VD - n (%)	2 (20%)	2 (%)		
Pre-operative PaO2/FiO2 ratio - Mean (SD)		371.05 (188.78)	475.24 (150.22)	0.36	0
Pre-operative Serum Creatinine (umol/L) - Mean (SD)		79.6 (10.62)	83.8 (22.35)	0.72	0
Pre-operative eGFR - Mean (SD)		75.08 (10.29)	89.24 (26.79)	0.32	0
Pre-operative Serum Troponin (ng/mL) - Mean (SD)		79.6 (10.62)	83.8 (22.35)	0.72	0
Pre-operative Serum NT-proBNP (pg/mL) - Mean (SD)		14 (16.2)	12 (13.6	0.79	0
Pre-operative MODS - Mean (SD)		0.8 (1.79)	0.8 (0.45)	1	0
Pre-operative Lactate - Mean (SD)		1.12 (0.62)	0.98 (0.32)	0.69	1
Pre-operative mean arterial pressure - Mean (SD)		94.06 (15.25)	103.46 (13.31)	0.33	0
Post-operative Lactate (at return to ICU) - Mean (SD)		2 (1.6 - 2.2)	1.9 (1.7 - 2.1)	0.75	0
Post-operative acute lung injury - Mean (SD)		1 (10%)	2 (%)	1	0
CBP time - Mean (SD)		131.6 (66.56)	119 (18.8)	0.7	0
Cross-clamp time - Mean (SD)		70 (39.93)	75.2 (16.77)	0.8	0

Table S5 – miR selection for verification with qRT-PCR.

miR	Source	Detected in urine
miR-10a-5p	This study	Yes
miR-26a-2-3p	This study	Not detected
miR-26a-2-5p	This study	Not detected
miR-196a-3p	This study	Not detected
miR-196a-5p	This study	Yes
miR-93-3p	This study	Not detected
miR-93-5p	This study	Yes
miR-99a-3p	This study	Yes
miR-99a-5p	This study	Yes
miR-103b	This study	Not detected
miR-21-3p	Saikumar et al., 2012, Du et al., 2013, Arvin et al., 2017	Not detected
miR-21-5p		Yes
miR-125a-5p	Sullo et al., 2018	Yes

1. Saikumar, J. *et al.* Expression, circulation, and excretion profile of microRNA-21, -155, and -18a following acute kidney injury. *Toxicol Sci* **129**, 256–267 (2012).
2. Du, J. *et al.* MicroRNA-21 and risk of severe acute kidney injury and poor outcomes after adult cardiac surgery. *PLoS One* **8**, e63390 (2013).
3. Arvin, P. *et al.* Early detection of cardiac surgeryassociated acute kidney injury by microRNA-21. *Bratisl Lek Listy* **118**, 626–631 (2017).
4. Sullo, N. *et al.* An Observational Cohort Feasibility Study to Identify Microvesicle and Micro-RNA Biomarkers of Acute Kidney Injury Following Pediatric Cardiac Surgery. *Pediatr Crit Care Med* **19**, 816–830 (2018).

Table S6 – Summary of miR comparisons between AKI and non-AKI.

q-value – p-values adjusted for multiple comparison using Benjamini Hochberg method.

Adjustment	Time	miR	Transformation	Normal distr	Test	p-value	q-value
None	Pre	miR-10a-5p	None	no	Wilcox	0.63	0.87
		miR-125a-5p	log	yes	ttest	0.07	0.53
		miR-196a-5p	None	no	Wilcox	0.95	0.95
		miR-21-5p	log	yes	ttest	0.66	0.87
		miR-320a-3p	log	yes	ttest	0.19	0.77
		miR-93a-5p	None	no	Wilcox	0.66	0.87
		miR-99a-3p	None	no	Wilcox	0.57	0.87
		miR-99a-5p	None	no	Wilcox	0.76	0.87
	Post	miR-10a-5p	log	yes	ttest	**0.02**	0.17
		miR-125a-5p	None	no	Wilcox	0.49	1.00
		miR-196a-5p	log	yes	ttest	0.81	1.00
		miR-21-5p	None	no	Wilcox	0.88	1.00
		miR-320a-3p	None	no	Wilcox	0.86	1.00
		miR-93a-5p	None	no	Wilcox	0.81	1.00
		miR-99a-3p	log	yes	ttest	1.00	1.00
		miR-99a-5p	None	no	Wilcox	0.42	1.00
Urinary particles concentrations	Pre	miR-10a-5p	log	yes	ttest	0.24	0.59
		miR-125a-5p	log	yes	ttest	**0.01**	0.11
		miR-196a-5p	None	no	Wilcox	0.40	0.59
		miR-21-5p	log	yes	ttest	0.53	0.59
		miR-320a-3p	log	yes	ttest	0.05	0.21
		miR-93a-5p	log	yes	ttest	0.59	0.59
		miR-99a-3p	None	no	Wilcox	0.35	0.59
		miR-99a-5p	None	no	Wilcox	0.45	0.59
	Post	miR-10a-5p	log	yes	ttest	**0.02**	0.20
		miR-125a-5p	None	no	Wilcox	0.41	0.75
		miR-196a-5p	log	yes	ttest	0.36	0.75
		miR-21-5p	None	no	Wilcox	0.32	0.75
		miR-320a-3p	None	no	Wilcox	0.47	0.75
		miR-93a-5p	None	no	Wilcox	0.80	0.92
		miR-99a-3p	log	yes	ttest	0.73	0.92
		miR-99a-5p	log	yes	ttest	0.95	0.95
Urinary creatinine	Pre	miR-10a-5p	log	yes	ttest	0.63	0.72
		miR-125a-5p	log	yes	ttest	0.22	0.72

		miR-196a-5p	None	no	Wilcox	0.54	0.72
		miR-21-5p	log	yes	ttest	0.35	0.72
		miR-320a-3p	log	yes	ttest	0.46	0.72
		miR-93a-5p	log	yes	ttest	0.55	0.72
		miR-99a-3p	None	no	Wilcox	0.83	0.83
		miR-99a-5p	log	yes	ttest	0.47	0.72
levels	Post	miR-10a-5p	log	yes	ttest	0.08	0.57
		miR-125a-5p	None	no	Wilcox	0.88	0.88
		miR-196a-5p	log	yes	ttest	0.63	0.84
		miR-21-5p	None	no	Wilcox	0.33	0.67
		miR-320a-3p	log	yes	ttest	0.52	0.84
		miR-93a-5p	None	no	Wilcox	0.33	0.67
		miR-99a-3p	log	yes	ttest	0.87	0.88
		miR-99a-5p	None	no	Wilcox	0.14	0.57

Table S7 – Summary of miR comparisons between AKI stages.

Post hoc comparisons (Tukey or Dunn tests for parametric and non-parametric data, respectively): S1-no – AKI stage 1 vs no AKI, S2+S3-no – AKI stage 2 or 3 vs no AKI, S2+S3-S1 – AKI stage 2 or 3 vs AKI stage 1.

Adjustment	Time	miR	Transformation	Normal	Test	p-value	S1-no	S2+S3-no	S2+S3-S1
None	Pre	miR-10a-5p	none	no	Kruskal	0.16	0.14	0.15	0.09
		miR-125a-5p	log	yes	ANOVA	**0.04**	**<0.05**	0.92	0.19
		miR-196a-5p	none	no	Kruskal	0.65	0.35	0.35	0.53
		miR-21-5p	log	yes	ANOVA	0.59	0.99	0.57	0.62
		miR-320a-3p	log	yes	ANOVA	0.43	0.42	0.81	0.99
		miR-93a-5p	none	no	Kruskal	0.52	0.48	0.40	0.22
		miR-99a-3p	none	no	Kruskal	0.33	0.25	0.19	0.25
		miR-99a-5p	none	no	Kruskal	0.84	0.45	0.83	0.46
	Post	miR-10a-5p	log	yes	ANOVA	0.06	0.14	0.16	0.84
		miR-125a-5p	none	no	Kruskal	0.74	0.66	0.46	0.53
		miR-196a-5p	log	yes	ANOVA	0.96	0.96	1.00	0.99
		miR-21-5p	none	no	Kruskal	0.25	0.22	0.17	0.15
		miR-320a-3p	none	no	Kruskal	0.97	0.45	1.00	0.66
		miR-93a-5p	none	no	Kruskal	0.79	0.47	0.36	0.78
		miR-99a-3p	log	yes	ANOVA	0.51	0.92	0.59	0.47
		miR-99a-5p	none	no	Kruskal	0.68	0.57	0.41	0.55
Urinary particles concentrations	Pre	miR-10a-5p	log	yes	ANOVA	0.17	0.24	0.84	0.30
		miR-125a-5p	log	yes	ANOVA	**0.02**	**0.02**	0.95	0.37
		miR-196a-5p	none	no	Kruskal	0.69	0.68	0.41	0.44
		miR-21-5p	log	yes	ANOVA	0.68	0.70	0.99	0.81
		miR-320a-3p	log	yes	ANOVA	0.16	0.17	0.55	1.00
		miR-93a-5p	log	yes	ANOVA	0.56	0.69	0.88	0.62
		miR-99a-3p	none	no	Kruskal	0.29	0.29	0.29	0.15
		miR-99a-5p	none	no	Kruskal	0.75	0.68	0.55	0.46
	Post	miR-10a-5p	log	yes	ANOVA	**<0.05**	0.22	0.06	0.49
		miR-125a-5p	none	no	Kruskal	0.70	0.71	0.41	0.44
		miR-196a-5p	log	yes	ANOVA	0.57	0.78	0.60	0.87
		miR-21-5p	none	no	Kruskal	0.58	0.45	0.54	0.38
		miR-320a-3p	none	no	Kruskal	0.54	0.36	0.40	0.30
		miR-93a-5p	none	no	Kruskal	0.41	0.23	0.28	0.29
		miR-99a-3p	log	yes	ANOVA	0.84	0.88	0.98	0.89
		miR-99a-5p	log	yes	ANOVA	0.72	0.96	0.78	0.70
Urinary	Pre	miR-10a-5p	log	yes	ANOVA	0.26	0.99	0.26	0.26

creatinine concentrations		miR-125a-5p	log	yes	ANOVA	0.10	0.18	0.71	0.17
		miR-196a-5p	none	no	Kruskal	0.64	0.37	0.53	0.36
		miR-21-5p	log	yes	ANOVA	0.41	0.85	0.38	0.60
		miR-320a-3p	log	yes	ANOVA	0.75	0.73	0.96	0.98
		miR-93a-5p	log	yes	ANOVA	0.52	0.97	0.49	0.59
		miR-99a-3p	none	no	Kruskal	0.37	0.25	0.22	0.25
		miR-99a-5p	log	yes	ANOVA	0.72	0.85	0.75	0.92
	Post	miR-10a-5p	log	yes	ANOVA	0.21	0.21	0.60	0.98
		miR-125a-5p	none	no	Kruskal	0.83	0.36	0.53	0.84
		miR-196a-5p	log	yes	ANOVA	0.69	0.98	0.67	0.76
		miR-21-5p	none	no	Kruskal	0.08	0.46	0.04	0.03
		miR-320a-3p	log	yes	ANOVA	0.82	0.85	0.90	1.00
		miR-93a-5p	none	no	Kruskal	0.60	0.48	0.51	0.40
		miR-99a-3p	log	yes	ANOVA	0.28	0.75	0.44	0.25
		miR-99a-5p	none	no	Kruskal	0.28	0.20	0.25	0.28
